# Effects of spray-dried plasma on performance, carcass parameters, tibia quality and Newcastle disease vaccine efficacy in broiler chicken fed corn-soy diets with two varying levels of digestible amino acids and AMEn density

**DOI:** 10.1371/journal.pone.0309263

**Published:** 2024-09-26

**Authors:** Atakan Bundur, Roshan Riaz, Fatma K. E. Elibol, Teyfik Demir, Javier Polo, Joe Crenshaw, Jürgen Zentek, Ozge Sizmaz

**Affiliations:** 1 Department of Animal Nutrition and Nutritional Diseases, Faculty of Veterinary Medicine, Ankara University, Ankara, Turkey; 2 Department of Biomedical Engineering, TOBB University of Economics and Technology, Ankara, Turkey; 3 Department of Mechanical Engineering, TOBB University of Economics and Technology, Ankara, Turkey; 4 APC Europe, S.L.U. Granollers, Granollers, Spain; 5 Department of Veterinary Medicine, Institute of Animal Nutrition, Freie Universität Berlin, Berlin, Germany; Kafkas University, TÜRKIYE

## Abstract

This study aimed to determine the effects of spray dried plasma (SDP) on growth performance, carcass traits, tibia quality, and hemagglutination inhibition titers in broilers fed two nutritional strategies with high or low nutrient density. In the study, 816 one-day-old Ross 308 male broiler chickens were divided into a 2 × 2 factorial arrangements consisting of four treatment groups with 12 replicates (17 birds/replicate) based on diets with high nutrient density (HND) or low nutrient density (LND) from d 0 to 42 and receiving either control or 1% SDP diets during d 0 to 10. The results showed that feed intake (FI) and body weight gain (BWG) were increased (P < 0.05) and feed conversion ratio (FCR) was significantly reduced (P = 0.003) for broilers fed HND diets from d 0 to 42. The inclusion of SDP increased the BWG (P < 0.001), FI (P < 0.001), and FCR (P < 0.05) during d 0 to 10 of broiler life but not effect of SDP was observed for the whole 0–42 d period. Carcass yield increased with HND (P < 0.001) and dietary SDP (P = 0.002). However, HND feeding significantly decreased liver (P < 0.001), bursa of Fabricius (P = 0.002), abdominal fat (P < 0.001), proventriculus (P < 0.001) and gizzard weight (P < 0.001), but increased heart weight (P = 0.013), although spleen weight remained unaffected (P > 0.05) on d 42. Tibial bone morphometric and mechanical properties improved (P < 0.05) with SDP supplementation, and bone ash, Ca, and P remained unaffected (P > 0.05) on d 14. With the exception at d 28 (P = 0.037), the antibody titer to ND virus was similar among all treatment groups (P > 0.05) at d 0, 14, and 42. In conclusion, HND diets improve performance of broilers during the whole period and SDP supplementation during starter phase improve performance at this period, but also increased carcass yield, and tibial quality. Therefore, inclusion of SDP in the starter diet could be a beneficial nutritional strategy to improve the health and production of broilers provided feeding strategies using various nutrient densities.

## Introduction

Growth rate of broilers has continually improved through genetic selection and improvement in nutrition and management conditions resulting in high growth rate with reduced number of days required to reach market weight [1]. However, fast growing broilers require a balanced and highly nutritious diet to show their maximum genetic potential and any modulation or deficiency in the diet can result in decreased performance, immunity, and economic return [2, 3]. Fast growing broilers are more susceptible to diseases, metabolic disorders, and skeletal problems [4, 5].

Studies have been performed with modifications in nutrient densities, including adjustments in dietary protein, amino acids, and energy to obtain better broiler performance [6]. However, the results of providing different nutrient densities in broiler diets are inconsistent. Barekatain and Swick [7] reported that higher amino acid (AA) density could improve bird performance because dietary amino acids activate components involved in translation initiation for proteins, and have a significant effect on protein synthesis. Many studies indicated that high AA-density diets improve body weight gain (BWG), feed conversion ratio (FCR), carcass weight and breast meat and reduce abdominal fat [8–11], whereas other studies reported no significant effects of nutrient density or feed restriction on performance, immune response, health, organs, and bone quality of broilers [12–14]. The effect of nutrient composition on early development becomes negligible when diets contain an adequate supply of essential nutrients and some studies also reported that higher nutrient density diets can increase feed costs [2, 15], diet density stress, metabolic problems [16], nitrogen excretion [17], foot lesions [18] and reduce economic return [2]. Any abnormality in the legs results in physiological weight loss, pain, gait problems, reduced feed, and water consumption, culling at the farm, and downgrading at processing, which overall increases welfare concerns and economic losses [16, 19–22]. Furthermore Zhai et al. [23] showed decreased feed intake (FI) and body weight (BW) of broilers fed low energy and high AA-density diets, but high energy and low AA-density diets increased the carcass parameters in birds raised in summer conditions. Other studies also indicated poor feed and production performance, low immune response, and increased occurrence of skeletal and gait issues in low nutrient density diet-fed birds compared to birds fed high nutrient density diets [18, 24–29].

Spray dried plasma (SDP) has gained the attention of researchers because of its nutritional and health benefits for animals and birds. The SDP contains immunoglobulins, peptides, albumins, enzymes, hormones, and nucleotides [30–32]. Spray dried plasma can enhance performance, nutrient digestibility, and gut health [33–35]. Under environmental stress, disease, and an unhygienic environment, several studies reported improvements in the immune response of birds fed SDP diets [33, 36–39]. A few studies showed a non-significant change in serum immunoglobulins in broilers fed SDP-supplemented diets [32, 33]. Early nutrition has been demonstrated to affect the development of the digestive and immune systems, resulting in increased growth in the following stages [40]. To the best of our knowledge, there are no studies on the effect of supplementing starter diets (d 1 to 10) with SDP on the performance, carcass characteristics, immune response and bone quality of broilers fed diets of different nutrient densities. Therefore, we hypothesize that supplementing the starter diet with SDP will reduce nutritional stress and improve performance, bone quality, and immune response in broilers regardless of the nutrient density of the diets.

The present study was designed to determine the effects of SDP, included only in the starter diet fed for 10 days, on growth performance, carcass, and organ weights as a percentage of live weight, tibia quality, and immune response to vaccination of broilers fed different nutrient densities over the entire 42-day study.

## Materials and methods

All animal care and experimental protocols were approved by the University of Ankara Animal Experiments Local Ethics Committee (2021-9-57).

### Experimental animals and diets

A total of 816 one-day-old Ross 308 male broiler chickens were obtained from the hatchery (Aviagen, Anadolu, Turkey). Individual chicks were weighed and randomly assigned to one of four experimental groups based on their initial body weight. Each group was represented by 12 replicates (pens) with 17 birds in each pen using a 2 × 2 factorial arrangement of dietary treatments. Dietary factors were 0% or 1% spray dried plasma (SDP) formulated into the starter diet fed from 0 to 10 days of age or two different nutrient density (ND) diets for all feed phases; low nutrient density (LND) diet with low digestible amino acid content and AMEn, or high nutrient density (HND) diet with high digestible amino acid content and AMEn. Birds were fed starter, grower1, grower2, and finisher diets from 0 to 10 d, 10 to 21 d, 21 to 35d, and 35 to 42 d of age, respectively (Table 1). In all experimental diets designed for each developmental phase, amino acids were calculated considering the dietary requirements suggested for Ross 308 (Table 2). Broilers were provided ad libitum access to feed in mash form and fresh water during the entire study. Birds were kept in floor pens with 8 to 10 cm deep layer of fresh wood shavings.

**Table 1:** Feed composition (as fed basis) with or without SDP in the starter diet and different nutrient densities per feeding period.

	Starter (d 0 to 10)				Grower 1 (d 10 to 21)		Grower 2 (d 21 to 35)		Finisher (d 35 to 42)	
Ingredient (%)	LND-SDP	LND-N	HND-SDP	HND-N	LND	HND	LND	HND	LND	HND
Corn	57.30	57.35	57.08	55.25	64.54	56.79	63.16	54.87	66.62	52.90
Soybean meal	37.48	37.81	28.54	28.98	24.04	21.24	13.85	11.54	9.51	4.33
Full fat soy meal	0.13	0.73	8.97	11.37	7.23	17.86	19.36	30.09	20.35	39.47
Mono-calcium phosphate	1.79	1.74	1.71	1.69	1.60	1.51	1.34	1.25	1.34	1.20
Calcium carbonate	1.17	1.17	1.21	1.20	1.15	1.14	1.04	1.02	1.06	1.04
Spray dried plasma (SDP)^1^	1.00	-	1.00	-	-	-	-	-	-	-
Na bicarbonate	0.40	0.37	0.40	0.40	0.44	0.44	0.42	0.40	0.43	0.37
DL-Methionine, 99%	0.24	0.26	0.34	0.36	0.29	0.34	0.23	0.28	0.18	0.25
NaCl	0.21	0.24	0.21	0.19	0.19	0.20	0.21	0.23	0.21	0.25
Choline chloride	0.10	0.07	0.09	0.07	0.11	0.08	0.11	0.09	0.13	0.09
Vitamin-Mineral premix^2^	0.10	0.10	0.10	0.10	0.10	0.10	0.10	0.10	0.10	0.10
L-Threonine	0.06	0.09	0.14	0.16	0.09	0.10	0.05	0.04	0.02	0.02
Lysine HCl	0.03	0.07	0.21	0.23	0.22	0.20	0.13	0.09	0.11	0.04

^1^ Spray-dried plasma (AP820™, APC Europe, S.L., Granollers, Spain)

^2^ Vitamin-mineral premix, per kg: retinole acetate,13,000,000 (IU); cholecalciferol, 5,000,000 (IU); DL-α-tocopherol acetate, 80,000 (IU); vitamin-K3, 3,200 (mg); vitamin-B1, 3,200 (mg); vitamin-B2, 8,600 (mg); vitamin-B6, 4,500 (mg); vitamin-B12, 17 (mg); niacin, 65,000 (mg); calcium pantothenic acid, 20,000 (mg); folic acid, 2,200 (mg); D-biotin, 250 (mg); manganese, 120,000 (mg); iron, 20,000 (mg); zinc, 110,000 (mg); copper, 16,000 (mg); iodine, 1,250 (mg); selenium, 300 (mg)

SDP = spray dried plasma; LND = low nutrient density diet; HND = high nutrient density diet; LND-N = low nutrient density control diet; LND-SDP = low nutrient density diet with 1% SDP; HND-N = high nutrient density control diet SDP; HND-SDP = high nutrient density diet with 1% SDP in starter diet.

**Table 2:** Analyzed and calculated nutrient and energy concentrations of high and low nutrient density diets (as fed basis) with or without 1% SDP in the starter diet.

	Starter (d 0 to 10)				Grower (d 10 to 21)		Grower (d 21 to 35)		Finisher (d 35 to 42)	
Nutrients	LND-N	LND-SDP	HND-N	HND-SDP	LND	HND	LND	HND	LND	HND
**Analyzed (%)**										
Dry Matter	89.13	89.13	89.34	89.56	90.84	90.10	89.83	90.23	89.48	89.82
Crude Protein	21.18	20.93	20.30	20.08	18.27	20.42	17.22	19.72	17.84	20.23
Ether Extract	2.23	2.01	3.71	4.34	3.66	5.21	6.55	7.92	7.21	9.34
Crude Fiber	4.20	4.08	3.86	4.03	3.31	3.62	3.85	3.95	3.40	3.44
Crude Ash	5.82	6.78	6.86	6.80	7.10	5.57	4.91	5.92	6.21	6.52
**Calculated**										
Metabolizable Energy (kcal/kg)*	2786	2732	2800	2843	2876	3029	2983	3069	3071	3152
Lys	1.15	1.15	1.24	1.24	1.05	1.15	0.95	1.05	0.85	1.00
TSAA	0.87	0.87	0.94	0.94	0.82	0.90	0.74	0.82	0.66	0.78
Thr	0.78	0.78	0.84	0.84	0.68	0.75	0.62	0.68	0.55	0.65
Val	0.96	0.96	0.92	0.92	0.79	0.86	0.76	0.84	0.70	0.83
Ile	0.85	0.85	0.82	0.82	0.71	0.79	0.69	0.77	0.63	0.76
Trp	0.18	0.18	0.23	0.23	0.19	0.20	0.17	0.19	0.15	0.18
Na	0.24	0.24	0.20	0.20	0.20	0.20	0.20	0.20	0.20	0.20
Ca	0.90	0.90	0.84	0.84	0.84	0.84	0.76	0.76	0.76	0.76
Available P	0.45	0.45	0.42	0.42	0.42	0.42	0.38	0.38	0.38	0.38

SDP = spray dried plasma; LND = low nutrient density diet; HND = high nutrient density diet; LND-N = low nutrient density control diet; LND-SDP = low nutrient density diet with 1% SDP; HND-N = high nutrient density control diet; HND-SDP = high nutrient density diet with 1% SDP in starter diet.

^*^Energy was calculated by the results of nutrient analysis

### Growth performance of the birds

Body weight and feed intake per pen were recorded on d 0, 10, 21, 35, and 42 and determined for each period and cumulatively. Mortality was recorded daily, and mortality corrected feed conversion ratio (cFCR) was calculated by adding dead bird weight. A correction was also made for the birds intentionally sacrificed via decapitation on day 0 and 14. Accordingly, the total weight of birds (live and dead) and the feed intake were multiplied by a factor (bird number on day 0)/ (bird number on day 0–birds intentionally sacrificed) [41].

### Carcass yield and organs relative weight

On day 42, three birds from each replicate were randomly selected for recording carcass and organ weights. After weighing, the birds were slaughtered in a clean slaughtering room by decapitation. Birds were de-feathered, eviscerated, and the empty carcass was weighed. The gallbladder was separated from the liver and the internal gizzard content was removed. The organs were weighed individually and carcass yield and organ weights were expressed as a percentage of body weight [42].

### Bone quality analysis

One randomly selected bird from each pen was sacrificed on day 14, and their tibias were removed to determine tibia strength, ash, Ca, and P content. After collection, tibia bones were cleaned and frozen at –20°C. After thawing, bones were weighed, and length, lateral cortex thickness, and proximal head thickness at the femoral and metatarsal sides were measured using a digital caliper (Yamayo, Tokyo, Japan) [43]. To calculate bone strength, tibias were subjected to the three-point bending tests until failure occurred, where tests were performed on Instron 5944 testing frame (Instron, Norwood, MA, USA). The loading rate was 5 mm/min. Spon length (**L**) was 30 mm. Load was applied at the midpoint of the shaft. Load vs. displacement data was collected for each specimen. Stiffness values were calculated from the load displacement curves slope of the linear region. Ultimate load and displacement at ultimate load were also determined from the load displacement curves. Yield load is the load which permanent deformation of the system begins. Displacement at yield load is the displacement at which permanent deformation begins. The tibia ash and mineral content was determined by the procedure described by [44]. According to the protocol, the tibia bone dehydration was done with ethanol for 72 h. The dehydrated bones were subjected to defatting for 72 hours in 9:1 diethyl ether: methanol solution respectively. After drying for 24 hours at 105°C, the tibias were ashed for 12 hours at 600°C. The ash content of the tibia was calculated as g/100 g fat free dry matter. Tibia ash was analyzed for Ca and P by using spectrophotometry [45].

### Serum collection and hemagglutination inhibition analysis

At d 0, blood samples were collected via decapitation from 15 randomly selected chicks (unvaccinated from the hatchery). This was used as the baseline of maternal titers. After arrival on day one, all chicks were subjected to Newcastle disease vaccine. Blood samples were collected from 2 birds per replication for analysis via the slaughtering process on the d 0 (vaccinated chicks from the hatchery), 14, 28, and 42. Antibody titers to ND virus were assessed by hemagglutination inhibition as described by Numan et al. [46].

### Statistical analysis

The data were analyzed using SPSS 14.01 program (SPSS Inc. Chicago, IL, USA) by two-way ANOVA using the GLM procedures. The analysis included the 2 x 2 factorial design for the main effects and interactions of the 2 different nutrient densities (LND vs HND) and the SDP supplementation (0% vs 1%) in the starter diet. The Duncan’s Multiple Range Test was used for the comparison of means. Statements of significance were based on *P*-value equal to or less than 0.05.

## Results

The performance parameters of the birds fed SDP by different ND diets are presented in Table 3. In the present study, no interaction was observed between ND and SDP for FI and FCR (P > 0.05). A significant interaction was found between ND and SDP for BWG at d 0 to 10 (P = 0.001). The highest BWG was for birds fed the HND-SDP diet at d 0 to 10 (P < 0.001). Whereas a non-significant (P > 0.05) interaction between ND and SDP for BWG was observed (Table 3) at other growth intervals (days 10 to 21, 21 to 35, 35 to 42, 0 to 42).

**Table 3:** Body weight gain, feed intake and corrected feed conversion ratio of broilers fed SDP in the starter diets at different nutrient densities.

Item		Body Weight Gain (g)					Feed Intake (g)					Corrected Feed Conversion Ratio (g:g)				
		0–10	10–21	21–35	35–42	0–42	0–10	10–21	21–35	35–42	0–42	0–10	10–21	21–35	35–42	0–42
LND	LND-N	144.74^b^	354.97	994.08	671.17	2089.20	271.22	784.90	1828.06	1060.80	3944.98	1.64	1.75	1.57	1.15	1.77
	LND-SDP	146.25^b^	371.65	1014.59	609.15	2141.64	310.38	774.44	1880.30	1143.61	4108.74	1.86	1.67	1.58	1.23	1.78
HND	HND-N	144.61^b^	446.32	1041.04	643.20	2275.16	270.17	876.03	1839.27	1127.59	4113.06	1.60	1.67	1.45	1.14	1.66
	HND-SDP	161.28^a^	438.90	1052.91	599.77	2252.86	307.21	838.42	1902.50	1123.99	4172.13	1.70	1.53	1.48	1.16	1.68
Main effects																
ND	LND	145.50^b^	363.30^b^	1004.33^b^	640.16	2115.42^b^	290.80	779.67^b^	1854.18	1102.20	4026.86^b^	1.75	1.71^a^	1.57^a^	1.19	1.78^a^
	HND	152.95^a^	442.61^a^	1046.97^a^	621.48	2264.01^a^	288.69	857.23^a^	1870.88	1125.79	4142.59^a^	1.66	1.60^b^	1.46^b^	1.15	1.67^b^
SDP	0%	144.68^b^	400.64	1017.56	657.18	2182.18	270.69^b^	830.47	1833.66^b^	1094.19	4029.02^b^	1.63^b^	1.71^a^	1.51	1.15	1.72
	1%	153.77^a^	405.28	1033.75	604.46	2197.25	308.79^a^	806.43	1891.40^a^	1133.80	4140.43^a^	1.78^a^	1.60^b^	1.53	1.20	1.73
SEM		1.452	6.705	8.355	13.778	23.144	4.643	9.709	12.757	13.592	28.089	0.027	0.024	0.016	0.018	0.018
P-value																
P -ANOVA		<0.001	<0.001	0.051	0.241	0.008	<0.001	<0.001	0.130	0.143	0.023	0.005	0.010	0.004	0.256	0.028
P -ND		0.001	<0.001	0.010	0.495	0.001	0.784	<0.001	0.503	0.377	0.032	0.078	0.017	<0.001	0.300	0.003
P -SDP		<0.001	0.495	0.312	0.059	0.720	<0.001	0.135	0.024	0.141	0.038	0.005	0.018	0.513	0.133	0.721
P-Interaction		0.001	0.080	0.786	0.734	0.377	0.891	0.394	0.825	0.109	0.321	0.120	0.436	0.726	0.390	0.880

ND = nutrient density; SDP = spray dried plasma; LND = low nutrient density diet; HND = high nutrient density diet; LND-N = low nutrient density control diet; LND-SDP = low nutrient density diet with 1% SDP; HND-N = high nutrient density control diet; HND-SDP = high nutrient density diet with 1% SDP in starter diet

SEM = standard error of mean

Within column, different superscripts show significant difference (*P* < 0.05; 0.01; 0.001)

The BWG was significantly (P = 0.001) increased by HND during the entire study period (d 0 to 42). At d 0 to 10 only, SDP supplementation in the broiler diet significantly (P < 0.001) increased BWG, however BWG remained similar during other growth periods (P > 0.05), irrespective of SDP supplementation.

Feed intake was significantly increased (P < 0.001) at d 10 to 21 in HND fed birds compared to those fed LND. Cumulative FI (d 0 to 42) remained higher in birds fed HND compared to ones fed LND. Dietary supplementation of SDP in the broiler diet significantly increased FI at d 0 to 10, 21 to 35 (P < 0.001, 0.05, respectively) and cumulative (d 0 to 42) FI remained higher (P < 0.05) for broilers fed the 1% SDP diet compared to those fed the 0% SDP diet.

The FCR of birds fed HND at d 10 to 21, d 21 to 35, and d 0 to 42 showed a significant improvement (P < 0.05, 0.001, 0.01, respectively) compared to birds fed LND. The supplementation of SDP in the starter diet affected FCR during d 0 to 10 and 10 to 21. During the early growth phase (d 0 to 10), SDP supplementation increased (P < 0.01) FCR, however FCR significantly reduced (P < 0.05) during d 10 to 21. Whereas SDP supplementation did not affect FCR during other study periods or for the whole study.

In the present study, no interaction (P > 0.05) was found among ND and SDP supplementation on carcass yield and organ weights, however, both SDP supplementation and ND significantly influenced the carcass yield of the birds (Table 4). Carcass yield was significantly higher in birds fed HND (P < 0.001) and SDP (P < 0.01) supplemented birds compared to birds that did not receive the SDP at the starter period or received LND diets. Organ weights, excluding the spleen, were significantly different among the groups (Table 4). Relative organ weights (bursa of Fabricius, liver, gizzard, and abdominal fat) were significantly higher (P ≤ 0.001) in LND vs HND fed birds. There was a significantly higher (P = 0.028) heart weight in HND vs LND fed birds. The SDP supplementation significantly decreased liver and heart weight as a percentage of body weight (P < 0.05), whereas no effects (P > 0.05) of SDP supplementation were observed on the other studied organs.

**Table 4:** Carcass yield and relative organs weights of broilers (d 42) fed SDP in the starter diets at different nutrient densities.

Item		Carcass Yield (%)	Liver (%)	Heart (%)	Spleen (%)	Bursa (%)	Abdominal Fat (%)	Proventriculus (%)	Gizzard (%)
LND	LND-N	68.20	1.98	0.43	0.15	0.30	1.50	0.40	1.21
	LND-SDP	69.32	1.89	0.40	0.14	0.27	1.56	0.40	1.27
HND	HND-N	71.40	1.84	0.44	0.13	0.24	1.07	0.31	1.17
	HND-SDP	71.68	1.78	0.43	0.13	0.25	1.04	0.31	1.15
Main effects									
ND	LND	68.76^b^	1.94^a^	0.42^b^	0.14	0.28^a^	1.53^a^	0.40^a^	1.24^a^
	HND	71.54^a^	1.81^b^	0.43^a^	0.13	0.25^b^	1.06^b^	0.31^b^	1.16^b^
SDP	0%	69.80^b^	1.91^a^	0.43^a^	0.14	0.27	1.29	0.35	1.19
	1%	70.50^a^	1.84^b^	0.41^b^	0.14	0.26	1.30	0.36	1.21
SEM		0.164	0.017	0.003	0.003	0.006	0.032	0.005	0.011
P-value									
P -ANOVA		<0.001	<0.001	0.013	0.638	0.002	<0.001	<0.001	0.001
P -ND		<0.001	<0.001	0.028	0.126	0.001	<0.001	<0.001	<0.001
P -SDP		0.002	0.023	0.016	0.758	0.538	0.760	0.447	0.474
P-Interaction		0.059	0.588	0.313	0.697	0.093	0.338	0.739	0.080

ND = nutrient density; SDP = spray dried plasma; LND = low nutrient density diet; HND = high nutrient density diet; LND-N = low nutrient density control diet; LND-SDP = low nutrient density diet with 1% SDP; HND-N = high nutrient density control diet; HND-SDP = high nutrient density diet with 1% SDP in starter diet

SEM = standard error of mean

Within column, different superscripts show significant difference (*P* < 0.05; 0.01; 0.001).

Tibial bone quality parameters are presented in Table 5. There was no interaction (P > 0.05) of ND and SDP for all tibia quality parameters, excluding max load (Table 5), where results remained significant (P < 0.05). The birds fed HND demonstrated significantly higher tibia length, proximal head thickness at metatarsal and femoral side, and stiffness compared to LND fed birds. The birds fed LND diet showed higher displacement of yield of tibia bone (P < 0.01). Broilers fed the 1% SDP starter diet had a significantly higher tibia bone weight, length, lateral cortex thickness, proximal head thickness at metatarsal and femoral side, and displacement at yield compared to birds fed the 0% SDP diet. The ND significantly affected the ash content, and it was highest (P = 0.003) for HND fed birds compared to LND fed birds. However, SDP supplementation did not affect (P > 0.05) the ash content in the birds fed different ND diets. There were no significant differences in tibia Ca and P content among ND or SDP supplementation.

**Table 5:** Tibia bone strength, Ash, Ca, and P of broilers (d 14) fed SDP in the starter diets at different nutrient densities.

Item		Weight (g)	Length (mm)	Lateral cortex thickness (mm)	Proximal head thickness at metatarsal side (mm)	Proximal head thickness at femoral side (mm)	Maxload (N)	Stiffness (N/mm)	Yield load (N)	Displacement at yield (mm)	Ash (%)	Ca (%)	P (%)
LND	LND-N	1.60	50.85	3.36	11.70	9.45	33.12^b^	23.41	30.95	1.48	37.76	12.14	6.54
	LND-SDP	1.91	52.79	3.51	11.88	10.04	31.52^b^	26.75	30.22	1.21	38.06	12.89	6.35
HND	HND-N	1.78	52.28	3.41	12.16	9.88	31.90^b^	27.62	30.39	1.09	39.45	13.55	6.70
	HND-SDP	2.06	53.93	3.68	13.27	10.26	40.41^a^	35.60	37.03	0.93	39.66	12.69	6.86
Main Effect													
ND	LND	1.76	51.82^b^	3.44	11.79^b^	9.74^b^	32.32	25.08^b^	30.59	1.35^a^	37.91^b^	12.52	6.45
	HND	1.92	53.11^a^	3.54	12.71^a^	10.07^a^	36.16	31.61^a^	33.71	1.01^b^	39.55^a^	13.12	6.78
SDP	0%	1.69^b^	51.57^b^	3.38^b^	11.93^b^	9.67^b^	32.51	25.51^b^	30.67	1.28^a^	38.61	12.84	6.62
	1%	1.99^a^	53.36^a^	3.60^a^	12.58^a^	10.15^a^	35.97	31.18^a^	33.62	1.07^b^	38.86	12.79	6.61
SEM		0.044	0.285	0.035	0.130	0.074	1.145	1.197	0.978	0.052	0.266	0.248	0.100
P-value													
P -ANOVA		0.129	0.115	0.070	0.002	0.122	0.054	0.003	0.076	0.001	0.003	0.373	0.344
P -ND		0.077	0.033	0.166	0.001	0.030	0.100	0.005	0.123	0.001	0.003	0.240	0.098
P -SDP		0.002	0.004	0.007	0.018	0.002	0.136	0.015	0.145	0.031	0.622	0.923	0.946
P-Interaction		0.839	0.804	0.387	0.081	0.479	0.032	0.299	0.071	0.562	0.933	0.120	0.388

ND = nutrient density; SDP = spray dried plasma; LND = low nutrient density diet; HND = high nutrient density diet; LND-N = low nutrient density control diet; LND-SDP = low nutrient density diet with 1% SDP; HND-N = high nutrient density control diet; HND-SDP = high nutrient density diet with 1% SDP in starter diet

SEM = standard error of mean

Within column, different superscripts show significant difference (*P* < 0.05; 0.01; 0.001).

Newcastle disease virus hemagglutination inhibition (NDV-HI) titers are presented in Table 6. An interaction among ND and SDP was found for NDV-HI at day 28 of the study. At day 28, the NDV-HI titer was significantly higher (P < 0.05) in HND-N birds compared to other groups. In addition, the titer was significantly higher in HND fed birds compared to LND fed birds (P < 0.001). No difference among the NDV-HI titers at d 0, 14, and 42 was observed in both LND and HND fed broilers, irrespective of SDP supplementation. No interaction was observed between ND and SDP on NDV-HI titers at these evaluation periods.

**Table 6:** Serum ND virus HI titer (log 2) of broilers fed SDP in the starter diet at different nutrient densities.

Item		HI0	HI14	HI28	HI42
LND	LND-N	8.04	5.71	10.33^c^	10.08
	LND-SDP	7.83	5.79	10.75^bc^	10.04
HND	HND-N	8.08	6.17	11.33^a^	9.88
	HND-SDP	8.79	5.88	11.04^ab^	10.04
Main effects					
ND	LND	7.94	5.75	10.54^b^	10.06
	HND	8.44	6.02	11.19^a^	9.96
SDP	0%	8.06	5.94	10.83	9.98
	1%	8.31	5.83	10.90	10.04
SEM		0.153	0.099	0.091	0.065
P-value					
P -ANOVA		0.135	0.392	0.001	0.689
P -ND		0.102	0.175	<0.001	0.431
P -SDP		0.411	0.601	0.710	0.637
P -Interaction		0.133	0.347	0.037	0.431

HI0, HI14, HI28, HI42: Newcastle disease hemagglutination titer at 0, 14, 28 and 42 days

ND = nutrient density; SDP: SDP = spray dried plasma; LND = low nutrient density diet; HND = high nutrient density diet; LND-N = low nutrient density control diet; LND-SDP = low nutrient density diet with 1% SDP; HND-N = high nutrient density control diet; HND-SDP = high nutrient density diet with 1% SDP in starter diet

SEM = standard error of mean

Within column, different superscripts show significant difference (*P* < 0.05; 0.01; 0.001).

## Discussion

### Body weight gain, feed intake, and feed conversion ratio

Dietary nutrient density is one of several nutritional factors that significantly impact the growth and health of broiler chickens [27]. The present study revealed a non-significant interaction among ND and SDP supplementation in broilers for BWG, FI, and FCR at all studied intervals except for BWG which was found significant at d 0 to 10. The BWG was higher in the birds fed HND vs LND at d 0 to 42. Over the trial period (d 0 to 42), the FI was higher and FCR improved in HND fed birds. The HND fed birds showed significantly higher FI and lower FCR compared to LND fed birds, during d 10 to 21 and d 10 to 21 and 21 to 35, respectively. These results agree with [27] findings that high nutrient density diets increased BW and FI and reduced FCR compared to broilers fed low nutrient density diets. Brickett et al. [24] reported higher body weight and improved FCR in high nutrient density diet fed birds than those fed low nutrient density diet. However, the FI reported was higher in the low-density diet fed birds.

Contrary to our results, Hamungalu et al. [26] reported no effect of the nutrient densities on BWG and FI from d 1 to 21 and higher FI in birds fed a LND diet from d 22 to 35 and d 1 to 35, although no change in BWG was observed during these periods. The FCR results remained in line with our findings. Previous research has shown that reducing nutrient density increases FI [13]. In some studies, no difference has been found in FCR and FI results in the broilers fed different dietary nutrient densities [47, 48]. Variations in results of broilers fed different nutrient density diets can be due to many reasons. Birds have the capacity to adjust their FI in response to various diet nutrient density dilutions [49]. Birds fed low nutrient density diets increase their feed intake to get the required nutrients for normal physiological functions and growth. Thus, they compensate for the weight gain through increased feed intake and obviously at the cost of higher FCR. However, similar to the results reported by Li et al. [27] the broilers fed HND diets resulted in higher BWG in our study. The increased body weight of birds given high-nutrient density diets is due to increased FI. The improved FCR in HND diet fed birds was achieved because the increase in body weight was more significant than the increase in the FI. Thus, within a specific range of nutrient density, increasing FI might result in a rise in BWG [27]. In addition, a suitable formulation of a high-density diet also has higher digestibility of nitrogen, fat, Ca, P [26], starch, and metabolizable energy utilization [47] based on the composition, feed forms, and raising conditions [18, 24].

Supplementation of SDP in the diet significantly increased the BWG and FI during d 0 to 10 compared to birds fed the starter diet without SDP supplementation. The FCR was higher during d 0 to 10, and it was lower during 10 to 21 respectively in the birds fed the SDP supplemented starter diet. Similar results were reported by Henn et al. [39], where the inclusion of SDP in the diet improved the growth performance of the birds in the early stages of life. Fasina et al. [50] also reported improved BWG and FCR in the early growth period of the birds fed SDP. In another experiment, Campbell et al. [38] reported that the birds provided a SDP diet had improved BWG, FI, and feed efficiency. Inclusion of SDP in poultry diets under normal raising conditions has been reported to did not affect BWG, FI [40, 51], and FCR during all growth phases [31]. On the contrary, Dabbou et al. [52] reported growth performance improved in the birds fed 2% SDP in the starter diet and 1% SDP in grower and finisher diets, respectively [52]. Belote et al. [40] also reported an improvement in FCR of birds fed 1% SDP compared to the control birds during their early development. Furthermore, SDP addition in the poultry diets under pathogenic environments and disease stress improves broiler growth rate, FI, and feed efficiency [33, 51, 53]. Owing to these previous studies, when raising broilers kept in stressed or unhygienic settings, SDP inclusion strengthens the immune system response through immunoglobulins and/or other functional proteins inherent in SDP, which could account for the higher growth rates in antigen or stress exposed birds. However, in the present study under normal raising conditions, SDP supplementation improved bird BWG during the early stage of development but, supplementation of SDP only in the starter feed (day 0 to 10) was not sufficient to bring changes in future performance under different nutrition density feeding conditions. In this context, the discontinued supplementation of SDP did not affect gut morphology and histopathology [52] or improve performance under normal raising conditions. Here, one can conclude that during the growing and finishing phases, the nutrient supply was sufficient and nutritional stress was not enough to bring changes in overall bird performance to 42 day of age, but supplementing SDP in feed in growing and finishing phases may maintain or improve the birds health and performance.

### Relative carcass yield and organ percentages

In the present study, HND feeding increased the carcass yield compared to LND-fed birds. This increase can be related to the high nutrient utilization and performance of birds given HND versus LND diets. In line with the present results, Brickett et al. [24] reported an increase in the carcass weight of birds fed a HND diet versus those fed a LND diet. However, no effect of ND on carcass yield was observed. The non-significant impact on carcass yield in broilers fed different ND has been reported in other studies [18, 27]. The SDP inclusion in the starter diet improved carcass yield of birds fed different ND diets and it was higher for birds fed HND diets. In one study, carcass yield remained similar among 42-d old broilers fed varying concentrations of SDP in the diet [39]. In another study, researchers reported a quadratic effect of the varying inclusion levels of SDP on the carcass weight [51].

This study results showed an increase in liver, bursa of Fabricius, abdominal fat, proventriculus and gizzard weight and a decrease in heart weight of birds fed LND diets compared to HND-diets. No difference was observed in the spleen weight percentage of the birds fed different nutrient density diets. Some studies reported no feed ND effects on the liver, gizzard, legs, and abdominal fat weight of the birds fed at low and high densities diets [13, 26]. Using the amount of abdominal fat as a guide for overall carcass fat content, the results in our study showed HND fed birds had lower relative abdominal fat. Lemme et al. [54] found that increasing protein content in broiler feeds reduced abdominal fat. Abdominal fat was significantly enhanced by high nutrient density in the study of Li et al. [27]. The difference in abdominal fat results from other studies shows the change in liver metabolism and accumulation of the fat in the abdomen. Similar to our study, Heckert et al. [55] reported an increase in bursa weight with a low nutrient density diet. In the present study, spleen weight remained unaffected by the nutrient densities which reflects low stress of the nutrient densities on the immune system and welfare of the birds.

The supplementation of SDP in the starter diet decreased the liver and heart weight and no effect was observed on the other organs. Similar to our findings, Dabbou et al. [52] reported no effect of the SDP supplementation on the spleen and bursa. Similarly, Beski et al. [33] also found a non-significant impact of SDP supplementation on bursa and thymus but an increase in bursa weight of broilers fed diets with SDP. This inconsistency in results can be due to the immune defense system response of birds reared under stress and unhygienic conditions to fight off disease-causing organisms.

### Bone strength and mineralization

Our results on the bone quality parameters show a non-significant relationship between ND and SDP in broilers, excluding max load, where it increased in birds fed the HND-SDP starter diet. Birds fed HND had increased tibial bone length, proximal head thickness at metatarsal and femoral side, and stiffness. The ash content of the tibial bone increased, which also showed a decrease in the displacement at yield of bone with HND compared to LND feeding; however, Ca and P content of both groups remained similar. Bruno et al. [56] results align with our results, which showed that restricted feeding did not change tibial bone weight while the tibial bone length was reduced with restricted feeding compared to ad libitum feeding. However, Brickett et al. [24] found no difference ash content of bones of birds fed different diet nutrient densities. These differences between studies can result from the availability of the nutrients and mineralization of the bones.

The supplementation of SDP in the starter diet improved tibial bone weight and other quality parameters, although displacement at yield remained lower in the SDP-supplemented birds. The ash, Ca, and P remained unchanged in tibial bone in both SDP supplemented and non-supplemented groups. No previous studies are available to compare the results of early SDP supplementation on the tibial bone quality in the broilers fed different nutrient density diets. One study by Ruff et al. [57] showed the tibial bone quality of SDP supplemented birds under heat stress and, similar to our findings, reported an increase in the tibial strength in heat-stressed birds, and ash content of tibial bone remained similar in no heat stress birds compared to the tibial ash of heat stress birds. Ruff et al. [33] found no change in the plasma Ca concentration of SDP-fed birds compared to a control group. Jamroz et al. [58] also reported non-significant effects of porcine blood products on the retention of Ca and P in the body. Bone mineralization cannot keep up with the rapid development of bones and shows a low mineralization profile during the early stages of bone formation [59]. Thus, supplementation of SDP in the starter period assists the development of good quality and solid tibial bone without changing the ash content of the bones at the final stages.

### Antibody titer of ND-virus

Nutrition plays a vital role in developing the immune response in broiler birds [60]. However, high diet nutrient density does not always result in good health and welfare of the birds [61]. In this study, NDV-HI titer remained unaffected by different nutrient densities irrespective of SDP inclusion in the diets at d 0, 14, and 42, except for d 28, where the interaction between ND and SDP found significant. In line of present study results, Guob et al. [12] also reported non-significant changes in the humoral and cell-mediated immunity in broilers fed different ND diets. Several studies reported no change in the immune organs and immune response with restriction of feed in the birds [61, 62]. Nabizadeh et al. [63] showed an increased immune response of birds fed a high ND diet compared to a control. Blue et al. [36] study showed an increase in NDV-titer with SDP supplementation in the diet compared to control. Several studies show that SDP assists the immune system functions in high stress and disease conditions [33, 39, 50]. Thus, the immune system response to different nutrient densities and SDP supplementation varies based on the bird’s deficiency of nutrient uptake and degree of stress.

## Conclusion

This study shows a non-significant interaction between different nutrient densities and SDP inclusion in the starter phase diet except for BWG at d 0 to 10. Feeding broilers with high nutrient density diets improves body weight gain, feed intake, feed conversion ratio, and carcass yield. The inclusion of SDP in the starter diet increases the performance during the starter phase and had effects on carcass yield, and tibia bone quality of the birds. This is the first definitive study reporting the effect of starter diet inclusion of SDP in broilers fed different nutrient density diets. The results support the potential utilization of SDP in feed for broilers provided different nutrient density diets and further investigations on continuous feeding of various dietary inclusion levels of SDP and its potential positive effects on nutrient digestibility, bird performance, gut health, and immunological response.
